# Correlates of depressive symptoms among Latino and Non-Latino White adolescents: Findings from the 2003 California Health Interview Survey

**DOI:** 10.1186/1471-2458-7-21

**Published:** 2007-02-21

**Authors:** Rafael T Mikolajczyk, Maren Bredehorst, Nadia Khelaifat, Claudia Maier, Annette E Maxwell

**Affiliations:** 1 School of Public Health, Department of Public Health Medicine, University of Bielefeld, Bielefeld, Germany; 2 School of Public Health and Jonsson Comprehensive Cancer Center, Division of Cancer Prevention & Control Research, University of California, Los Angeles, 650 Charles Young Dr. South, Room A2-125, CHS, Los Angeles, CA 90095-6900. USA

## Abstract

**Background:**

The prevalence of depression is increasing not only among adults, but also among adolescents. Several risk factors for depression in youth have been identified, including female gender, increasing age, lower socio-economic status, and Latino ethnic background. The literature is divided regarding the role of acculturation as risk factor among Latino youth. We analyzed the correlates of depressive symptoms among Latino and Non-Latino White adolescents residing in California with a special focus on acculturation.

**Methods:**

We performed an analysis of the adolescent sample of the 2003 California Health Interview Survey, which included 3,196 telephone-interviews with Latino and Non-Latino White adolescents between the ages of 12 and 17. Depressive symptomatology was measured with a reduced version of the Center for Epidemiologic Studies Depression Scale. Acculturation was measured by a score based on language in which the interview was conducted, language(s) spoken at home, place of birth, number of years lived in the United States, and citizenship status of the adolescent and both of his/her parents, using canonical principal component analysis. Other variables used in the analysis were: support provided by adults at school and at home, age of the adolescent, gender, socio-economic status, and household type (two parent or one parent household).

**Results:**

Unadjusted analysis suggested that the risk of depressive symptoms was twice as high among Latinos as compared to Non-Latino Whites (10.5% versus 5.5 %, p < 0.001). The risk was slightly higher in the low acculturation group than in the high acculturation group (13.1% versus 9.7%, p = 0.12). Similarly, low acculturation was associated with an increased risk of depressive symptoms in multivariate analysis within the Latino subsample (OR 1.54, CI 0.97–2.44, p = 0.07). Latino ethnicity emerged as risk factor for depressive symptoms among the strata with higher income and high support at home and at school. In the disadvantaged subgroups (higher poverty, low support at home and at school) Non-Latino Whites and Latinos had a similar risk of depressive symptoms.

**Conclusion:**

Our findings suggest that the differences in depressive symptoms between Non-Latino Whites and Latino adolescents disappear at least in some strata after adjusting for socio-demographic and social support variables.

## Background

Depression is a very frequent health problem which is growing at an alarming rate. It has been suggested that if the trend persists, by 2020, depression will be the second biggest health care problem after heart disease worldwide [1]. The prevalence of depression is increasing not only among adults, but also among adolescents [2]. This increase is mostly related to the moderate forms of depressions without psychomotoric and physical symptoms [3]. Nevertheless, this form of depression is still strongly affecting health and quality of life of the patients. Population based studies show that at any one time between 10 and 15% of the child and adolescent population has some symptoms of depression [4]. Our paper focuses on symptoms of depression based on self-report.

Several risk factors for depression among adolescents have been identified, including female gender, increasing age, lower socio-economic status, and Latino ethnic background [5,6]. The effect of acculturation on depression has also been investigated. The term *acculturation *generally refers to the process whereby the attitudes and/or behaviors of persons from one culture are modified as a result of contact with a different culture. A recent literature review examined the association between acculturation and depression among Latinos. High acculturation was associated with a worse outcome in two studies, with a positive effect in one study, and had mixed or no effect on depression in three studies [7]. The author argues that past research was inconsistent in the measurement of acculturation or in the adjustment for possible confounding factors. In some cases, when studies have controlled for factors such as age, education or other factors, the effects of acculturation diminish or disappear [7]. In addition to demographic characteristics it has been suggested that social support and supervised care after school may be protective factors [8,9].

The aim of our study is to investigate the association between demographic factors (age, gender, ethnic background, socio-economic status), acculturation, social support and depressive symptoms in a large population-based sample of Latino and Non-Latino White adolescents residing in California.

## Methods

### Sample and variables

A secondary analysis was conducted using data from the adolescent sample of the 2003 California Health Interview Survey (CHIS) [10]. CHIS is a population-based, random-digit dial telephone survey which is representative for California's households in 2003. The analysis was based on 3,196 telephone-interviews with Latino and Non-Latino White adolescents between the ages of 12 and 17.

#### Measure of depressive symptoms

Depressive symptoms were measured according to a reduced version of the Center for Epidemiologic Studies Depression Scale (CES-D) following Radloff [11], who first adopted the CES-D Scale to children and adolescents. There were eight items in total, covering depressed affect (felt depressed, lonely, sad, could not shake off feeling sad and unhappy, felt life was a failure), happiness (were happy, enjoyed life), and retarded activity (did not want to do the things you usually do) during the past 7 days. Similarly to Radloff, the CHIS questionnaire did not ask for interpersonal aspects, because problematic peer relationships might be the norm for adolescents. There was a four point answer scale for the eight items (never, sometimes, a lot of the time, most of the time); for two of the items the scores were reversed, then the sum of the scores (0, 1, 2, 3) was calculated with higher scores indicating more symptoms.

Although the CES-D has been used as a screening instrument for depressive symptoms among adolescents, there are no established cutoff scores of the 8-item version of the CES-D Scale that was used in the CHIS. Cutoff scores of ≥ 8 and ≥ 10 have been used on the 10 item CES-D Scale in adult samples (e.g., [12]). Cutoff of ≥ 16 indicates depressive symptoms in the original 20 item CES-D (maximum score = 60 points), which was validated using DSM_III criteria. We set a cutoff score of >10 as an indictor for depressive symptoms for the 8 item CES-D (maximum score = 24) in the CHIS sample, which corresponds to a cutoff score of >25 on the 20 item scale. Based on analyses by Roberts et al., [13] 25 is the midpoint in the "moderately depressed" category in a student sample that completed the 20-item CES-D.

Few studies have assessed psychometric properties of the CES-D Scale among adolescents from diverse ethnocultural groups [14-16]. These studies suggest that it is appropriate to use the CES-D among Mexican-American adolescents, since their depression symptomatology is very similar to that of their Anglo-American peers. In our sample, factor analysis yielded identical factor structure and similar factor loadings in all three groups. High internal reliability indicated high homogeneity of the scale: Cronbach's alpha of the 8-item CES-D scale ranged from .73 in the low acculturation Latino subgroup to .79 among Non-Latino Whites.

#### Ethnic background and acculturation index

**Figure 1:**
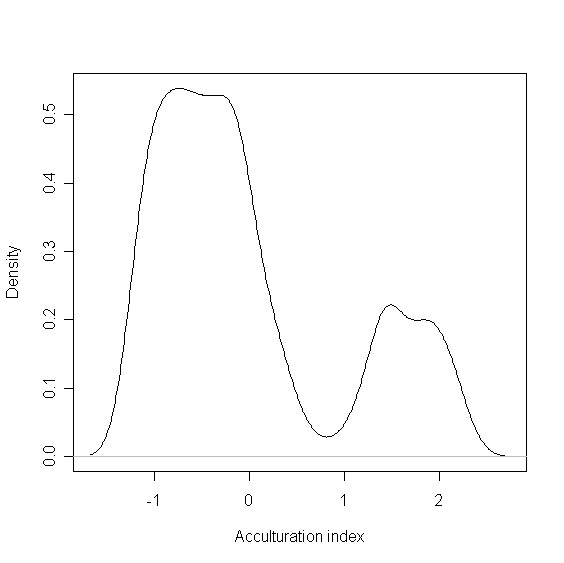
Distribution of the acculturation index (Kernel density plot).

Our analysis was based on data from Latino and Non-Latino White respondents. Within the Latino subgroup we computed an acculturation index based on the following variables: language in which the interview was conducted, language(s) spoken at home, place of birth, and number of years lived in the United States, citizenship status of the adolescent and both of his/her parents. The index was created using canonical principal component analysis (CAPCTA) [17], the nonparametric version of the principal component analysis, which has to be used when variables are either multinomial or ordinal. In a density plot the distribution of the acculturation index showed two clearly separated groups (see Figure 1.). Based on visual inspection, the index was dichotomized at 0.6 into high and low acculturation. The components of the acculturation index together with the categories of the acculturation index are presented in Table 1. The high acculturation Latino group was US born, bi-lingual and most of the interviews were conducted in English. The low acculturation Latino group was characterized by youth being foreign born and more frequent use of Spanish only.

**Table 1:** Ethnic, language and immigration characteristics by acculturation index

	Non-Hispanic White	Latino (high acculturation)	Latino (low acculturation)
Characteristics	N = 2,071 %	N = 865 %	N = 260 %

Country of birth			
US	96.9	99.5	
Other	3.1	0.5	100
Citizenship			
Native	96.9	99.5	
Naturalized	1.8	0.5	20.8
Other	1.3		79.2
Living in the US			
Born in the US	96.9	99.5	
10 or more years	1.4	0.5	43.5
5 – 9 years	0.9		25.0
2 – 4 years	0.5		22.7
1 or less years	0.3		8.8
Language at home			
English	85.6	24.3	1.5
English + other language	13.2	67.2	69.6
Spanish	1.2	8.6	28.8
Interview language			
English	99.7	87.4	47.3
Spanish	0.3	12.6	52.7
Citizenship and immigration status of mother			
Native	88.6	39.3	1.9
Naturalized	7.3	23.4	5.8
Other	4.2	37.3	92.3
Citizenship and immigration status of Father			
Native	89.3	34.5	3.8
Naturalized	6.7	26.9	14.6
Other	4.0	38.6	81.5

#### Other variables used in the analysis

Support provided by adults at school and at home was each measured with 8 items assessing the presence of adults who cared about, listened to and encouraged the adolescent respondent. Using CAPCTA, these variables were combined into two separates scores which were dichotomized at the median into low and high categories. The correlation between the support variables was below 0.4. Additionally we included in the analysis: age of the adolescent (in three groups 12–13, 14–15, 16–17), gender, socio-economic status (<200% poverty level and ≥ 200% poverty level), and household type (two parent or one parent household).

### Statistical analysis

Factor analysis was conducted to confirm identical factor structure of the CES-D scale for all subgroups. Internal reliability of the scale was tested by Cronbach's alpha. Univariate analysis was performed by tabulation and chi-square tests between Non-Latino White and Latino adolescents with high or low level of acculturation. We used the conventional significance level (p < 0.05). Univariate und multivariate logistic regression was used for the analysis of association between the risk of depressive symptoms and other variables. Multicollinearity test was performed based on the tolerance coefficient – no collinearity problems were detected for the analyzed variables. We also examined the effect of acculturation in the Latino subsample, using a continuous acculturation score (data not shown) and in a second step as a dichotomous variable. Finally, the possibility of effect modification between ethnic background and other variables was investigated. The modeling strategy followed Hosmer and Lemeshow [18]. All variables were included in a multiple logistic regression model. All two-ways interactions were investigated in separate steps with the main effects model. Interactions significant at the 0.05 level (based on Wald-test) were included jointly in the preliminary final model. The final model was obtained by removing effects which were not significant at the 0.05 level. For variables with effect modification the effect of ethnic background was calculated for different strata. All analyses were performed using SPSS 12.0.

## Results

### Description of the sample

The total sample consisted of 3196 adolescents: 2071 Non-Latino White youth, 865 Latino youth in the high acculturation category and 260 in the low acculturation category. The three groups were significantly different with respect to most socio-demographic characteristics (see Table 2). Latino youth came more frequently from lower income households, and reported lower support at home or at school. The low acculturation Latino group was the most disadvantaged in terms of social support and income. However, more Latino youth in the low acculturation group came from two parent households as compared to high acculturation Latinos and Non-Latino Whites.

**Table 2:** Socio-demographic characteristics by ethnicity and acculturation

	Non-Latino White	Latino (high acculturation)	Latino (low acculturation)
Characteristics	N = 2071 %	N = 865 %	N = 260 %

Age*			
12–13 years	32.7	40.5	31.5
14–15 years	33.9	33.6	31.5
16–17 years	33.3	25.9	36.9
Gender			
Male	50.4	51.1	54.2
Female	49.6	48.9	45.8
Poverty level*			
<200%	16.7	60.8	88.1
200% or more	83.3	39.2	11.9
Household Type*			
Both parents	75.0	72.6	83.8
One parent	25.0	27.4	16.2
Support at home*			
Low	44.5	57.8	63.8
High	55.5	42.2	36.2
Support at school*			
Low	42.1	56.2	65.4
High	57.9	43.8	34.6

*significant differences between the three subgroups, p < .001, chi-square test

### Characteristics associated with depressive symptoms

Scores on the 8-item CES-D Scale ranged from 0–24, with a mean score of 4.4 and standard deviation of 3.7. A total of 232 adolescents (7.3% of the sample) had scores >10 and were classified as having symptoms of depression (see Table 3). Based on univariate analysis, a significantly larger proportion of Latino youth had depressive symptoms as compared to Non-Latino Whites (10.5% versus 5.5%, p < 0.001). Although more low acculturation Latinos had symptoms of depression than high acculturation Latinos (13.1% versus 9.7%), this difference was not statistically significant (chi-square, p = 0.12). Females, youth living in households below 200% poverty level, those living in a one parent household and those who received low support at home and at school were significantly more likely to have symptoms of depression. Age was not associated with symptoms of depression in this sample.

**Table 3:** Characteristics associated with depressive symptoms in univariate and multivariate analysis (main effects only, N = 3,196)

Characteristics	Depressive Symptoms N (%)	chi-square p-value	Univariate OR (95% CI)	Multivariate* OR (95% CI)
Total sample	232 (7.3)		-	-
Ethnic background		<0.001		
Non-Latino White	114 (5.5)		1	1
Latino (high acculturation)	84 (9.7)		**1.85 (1.38–2.48)**	1.39 (1.00–1.95)
Latino (low acculturation)	34 (13.1)		**2.58 (1.72–3.88)**	**1.81 (1.12–2.93)**
Age		0.7		
12 – 13	75 (6.8)		0.92 (0.66–1.23)	0.96 (0.68–1.35)
14 – 15	83 (7.7)		1.06 (0.76–1.46)	1.10 (0.78–1.54)
16 – 17	74 (7.3)		1	1
Gender		<0.001		
Male	78 (4.8)		1	1
Female	154 (9.8)		**2.16 (1.63–2.86)**	**2.30 (1.72–3.07)**
Poverty Level		<0.001		
≥ 200%	116 (5.5)		1	1
<200%	116 (10.5)		**2.01 (1.54–2.63)**	1.34 (0.96–1.87)
Household Type		<0.001		
Both parents	150 (6.3)		1	1
One parent	82 (10.3)		**1.72 (1.30–2.28)**	**1.55 (1.15–2.09)**
Support at home		<0.001		
High	88 (5.5)		1	1
Low	144 (9.1)		**1.72 (1.31–2.27)**	1.16 (0.87–1.57)
Support at school		<0.001		
High	52 (3.1)		1	1
Low	180 (11.8)		**4.16 (3.03–5.71)**	**3.52 (2.52–4.92)**

*adjusted for all other variables in the table

In multivariate analysis, not accounting for effect modification, the impact of ethnic background and acculturation decreased as compared to univariate analysis (Table 3, columns 4 and 5). In this analysis, the odds of depressive symptoms were only increased among low acculturation Latinos as compared to Non-Latino Whites. Poverty level and low level of support at home no longer emerged as independent predictors of depressive symptoms. In this analysis, the most important predictors of depressive symptoms were low support at school, female gender, being classified as low acculturation Latino and coming from a one parent household.

### Characteristics associated with depressive symptoms in the Latino subsample

We examined independent predictors of depressive symptoms among Latinos by limiting the analysis to the Latino subsample. Similar to the multivariate analysis in the whole sample shown in Table 3, female gender and low support at school emerged as risk factors in the Latino subsample (Table 4). In the low acculturation group the odds of depressive symptoms were 50% higher than in the high acculturation group, but the effect did not reach statistical significance. There was no substantial change in effects of other variables after the inclusion of the acculturation variable in the joint model, only the impact of poverty level slightly decreased. There was also no evidence of effect modification in the Latino subsample which was assessed by significance of interaction terms (data not shown).

**Table 4:** Characteristics associated with depressive symptoms in the Latino subsample (N = 1,125)

	Model not including acculturation		Model including acculturation	
Characteristics	OR (95% CI)*	p-value	OR (95% CI)*	p-value

Acculturation low vs. high	-		1.54 (0.97–2.44)	0.07
Age: 12–13 vs. 16–17	1.22 (0.75–2.00)	0.4	1.30 (0.79–2.14)	0.30
Age: 14–15 vs. 16–17	1.21 (0.73–2.01)	0.5	1.26 (0.75–2.09)	0.39
Gender: Female vs. male	**2.60 (1.72–3.93)**	**<0.001**	**2.64 (1.74–3.99)**	**<0.001**
<200% poverty level vs. > 200%	0.96 (0.63–1.46)	0.8	0.86 (0.55–1.33)	0.50
Household type: One parent vs. both	1.29 (0.84–1.99)	0.2	1.37 (0.88–2.13)	0.16
Support at home: low vs. high	0.85 (0.57–1.29)	0.5	0.84 (0.55–1.27)	0.40
Support at school: low vs. high	**2.53 (1.60–4.00)**	**<0.001**	**2.50 (1.58–3.97)**	**<0.001**

*adjusted for all other variables in the table

### Ethnic differences in depressive symptoms in different strata of covariates

The comparison of the results in the Latino subsample (Table 4) and Latino and Non-Latino White combined sample (Table 3) revealed a strong effect modification. The poverty level, support at home and support at school had consistently a weaker association with depressive symptoms in the Latino sample than in the whole sample. This was confirmed in the formal analysis of interactions and the stratified results are presented in Table 5. The highest odds ratios were found in the more advantaged strata: the odds ratios for Latinos to have depressive symptoms as compared to Non-Latino Whites ranged from 2.1 in the strata with poverty level ≥ 200% to 3.29 in the strata with high support at school. The odds ratio of having depressive symptoms among Latinos as compared to Non-Latino Whites was 4.62 in the strata with high income and high support both at school and at home. The odds of having depressive symptoms were not different between Latinos and Non-Latino Whites in the strata that had lower levels of income and support at home. Predictors of depressive symptoms also varied somewhat in the male and female subsamples (Table 6). In both gender groups, low support at school remained the most important predictor of depressive symptoms. Among males, additional predictors were low support at home and coming from a one parent household which did not emerge as risk factors among females. Among females but not among males, low family income and being 14–15 years old emerged as risk factors for depressive symptoms. Ethnicity emerged as risk factor for depressive symptoms only among females.

**Table 5:** Ethnic differences in depressive symptoms in different strata of covariates

Characteristics	OR (95% CI)*
Within poverty level ≥ 200	
Non-Latino White	1
Latino	**2.10 (1.38–3.19)**
Within poverty level <200	
Non-Latino White	1
Latino	0.96 (0.62–1.50)
Within high support at home	
Non-Latino White	1
Latino	**2.75 (1.75–4.32)**
Within low support at home	
Non-Latino White	1
Latino	1.28 (0.90–1.82)
Within high support at school	
Non-Latino White	1
Latino	**3.29 (1.86–5.82)**
Within low support at school	
Non-Latino White	1
Latino	**1.41 (1.02–1.93)**
Within: poverty level≥ 200, high support at school and at home	
Non-Latino White	1
Latino	**4.62 (1.63–13.10)**

*all models include: age, gender, poverty level, ethnic background, support at home and support at school (where appropriate)

**Table 6:** Gender differences in predictors of depressive symptoms: Separate analysis for males and females

	Male (N = 2048)		Female (N = 1962)	
Characteristics	OR (95% CI)*	p-value	OR (95% CI)*	p-value

Latino versus Non-Latino White	1.27 (0.74–2.19)	0.39	**1.60 (1.08–2.39)**	**0.02**
Age: 12–13 vs. 16–17	1.00 (0.58–1.71)	0.99	0.92 (0.59–1.44)	0.71
Age: 14–15 vs. 16–17	0.56 (0.31–1.02)	0.06	1.51 (0.99–2.29)	0.06
<200% poverty level vs. > 200%	1.12 (0.64–1.94)	0.70	**1.54 (1.03–2.30)**	**0.04**
Household type: One parent vs. both	**2.36 (1.46–3.81)**	**<0.001**	1.16 (0.79–1.70)	0.45
Support at home: low vs. high	**1.73 (1.01–2.98)**	**0.05**	0.97 (0.68–1.39)	0.86
Support at school: low vs. high	**3.21 (1.80–5.72**)	**<0.001**	**3.78 (2.51–5.71)**	**<0.001**

* adjusted for all other variables in the tables

## Discussion

We conducted extensive analyses in a population-based sample of Latino and Non-Latino White adolescents to examine associations between depressive symptoms and socio-demographic variables (age, gender, ethnicity, income, one parent versus two parent household type), acculturation, and social support at home and at school. Crude analyses suggested that the risk of depressive symptoms was twice as high among Latinos as compared to Non-Latino Whites (10.5% versus 5.5 %). Other risk factors included female gender, low household income, one parent household, and low support at home and at school. All of these factors have been reported as risk factors for depressive symptoms among Latinos and other ethnic groups [6,8,9,19].

However, when all risk factors were considered simultaneously in a multivariate analysis, only four independent risk factors emerged: having low support at school, being female, being classified as low acculturation Latino and coming from a one parent household. In a stratified analysis, risk factors that were unique to males were low support at home and coming from a one parent household. Ethnicity was not a risk factor in this stratified analysis, suggesting that these risk factors are similar among both Non-Latino White and Latino male adolescents regardless of ethnic background. Almost one third of children less than 18 years of age in California (29%) live in one parent households: 21% live in mother only households and 7% in father only households [20]. Thus, boys are more likely than girls to live in a one parent household with a parent of the opposite gender. It may be that males growing up without a father in the household are either experiencing something or lacking something, such as for example a male role model, that increases their risk of depressive symptoms. Patten and colleagues [8] analyzed data of a large sample of California adults and also found higher rates of depressive symptoms among adolescents living in one parent households than in those living in two parent households. Their study showed highest rates of depressive symptoms among girls living in father only households (25.1% vs. 19.35 in mother only households), whereas the rates of depressive symptoms for boys were around 16% for both father or mother only households [8]. The relative effect of single parent household (which are predominantly single mother households) was stronger for boys than for girls in our analysis. Further analyses by Patten and colleagues [8] revealed that household type has to be considered in conjunction with parental support, as even in a two parent household risk of depression was increased if the adolescents perceived that they were not able to talk to either parent about their problems. Clearly, the complex relationship between depression, household type and parental support and the mechanisms of how these variables may relate to depression need to be further studied.

Risk factors of depressive symptoms that were unique to females in our sample were Latino ethnicity, age 14–15 and low household income. Latino females emerged as risk group for depressive symptoms in both a gender stratified analysis and in an analysis limited to the Latino subsample. Interestingly, the age group 14–15 years had the highest risk of depressive symptoms among females, but the lowest risk for males. Thus, in the combined analysis, the risk estimates were averaged and age did not emerge as a risk factor for depressive symptoms. Our findings suggest that boys and girls show different profiles of correlates and probable risk factors for depressive symptoms. Others have suggested that risk factors for depression such as stress and social support may have a greater impact among girls than among boys [9]. Future studies need to further evaluate gender differences in rates and risk factors of depression as gender specific intervention programs may be needed.

In our sample, low support at school was the strongest risk factor for depressive symptoms for both males and females. This variable captured respondents' perceptions of the availability of a teacher or other adult at school who "noticed when they were not there, listened to them when they had something to say, told them when they did a good job, always wanted them to do their best, and noticed when they were in a bad mood". Thus, teachers and school counselors are important sources of support, and need to be trained to recognize symptoms and risk factors of depression. They also need to be given the time to pay attention to individual students.

A multivariate analyses taking into account existing interactions between socio-economic status, perceived support and ethnicity provided a profile of depressive symptoms that was even more detailed. When we examined different strata of household income and support, either at home or at school, Latino ethnicity emerged as risk factor for depressive symptoms only among the strata with higher income and high support at home and at school. While this finding is counterintuitive at first, it suggests that high economic status and social support are protective factors only among Non-Latino Whites. We have not been able to find any literature that is investigating this hypothesis. An alternative interpretation relates to the association between depressive symptoms and perceived discrimination. Several studies suggest that higher income is associated with more perceived discrimination and that discrimination is a risk factor for depression [21,22]. Since CHIS does not assess perceived discrimination, we were not able to examine this relationship. We found no ethnic differences between Latino and Non-Latino Whites in the prevalence of depressive symptoms in the strata with low income or low social support at home

Although our data suggest several correlations between socio-demographic characteristics, social support and depressive symptoms, the causal nature of these relationships is ambiguous given the cross sectional study design. As pointed out by others [8], depressed adolescents may be less inclined to form supportive relationships with parents, teachers or peers, and less likely to perceive relationships as supportive, and to report supportive relationships. Another limitation of our data set is that several variables that have been shown to be risk factors for depression, such as stressful life events [9], perceived discrimination and low self esteem [21-23], being involved in bullying either as a perpetrator or as a victim [5], affiliation with high-versus low status peer crowd, negative or positive qualities of friendships, and presence or absence of romantic relationships [24] were not available. Finally, as in many other studies, our measure of acculturation may not have captured aspects of the acculturation process that are related to depression. Although we attempted to include all data related to the acculturation experience that were available in this data set in developing an acculturation scale, and although we used a method that has the advantage of not making inappropriate statistical assumptions, the dichotomized acculturation variable that we created was almost identical to a simple dichotomization based on country of birth (US versus other). Finally, our sample of low acculturation Latino respondents was relatively small and given that most Latinos living in California are from Mexico, findings may not be generalizable to those with different heritage. However, despite these limitations, our analysis adds some information to the sparse literature on depression among ethnically diverse adolescents.

## Conclusion

Our findings suggest that differences in depressive symptoms between Non-Latino Whites and Latino adolescents disappear at least in some strata after adjusting for socio-demographic and social support variables and gave rise to some interesting hypotheses regarding modifiers of depression such as household income, social support and gender. These hypotheses should be further investigated in order to identify groups that are at high risk for depression and could benefit from interventions.

## Competing interests

The authors declare that they have no competing interests.

## Authors' contributions

RTM analyzed the data and participated in the interpretation of the results and the writing of the final version of the manuscript. MB, NK, CM developed the research question, performed preliminary analyses and wrote a few sections of the manuscript as part of a class assignment. AEM participated in the interpretation of the results and wrote large parts of the final version of the manuscript. All authors approved the final version of the manuscript.

## Pre-publication history

The pre-publication history for this paper can be accessed here:


